# International transfers of personal data for health research following Schrems II: a problem in need of a solution

**DOI:** 10.1038/s41431-021-00893-y

**Published:** 2021-05-06

**Authors:** Dara Hallinan, Alexander Bernier, Anne Cambon-Thomsen, Francis P. Crawley, Diana Dimitrova, Claudia Bauzer Medeiros, Gustav Nilsonne, Simon Parker, Brian Pickering, Stéphanie Rennes

**Affiliations:** 1 FIZ Karlsruhe—Leibniz-Institut für Informationsinfrastruktur, Hermann-von-Helmholtz-Platz 1, Eggenstein-Leopoldshafen, Germany; 2 McGill University Faculty of Medicine, Centre of Genomics and Policy, Montreal, Canada; 3 CNRS, Center for Epidemiology and Research in POPulation health (CERPOP), Université de Toulouse, Inserm, UPS, Joint Unit 1295, Toulouse, France; 4 Good Clinical Practice Alliance—Europe (GCPA) and Strategic Initiative for Developing Capacity in Ethical Review (SIDCER), Leuven, Belgium; 5 FIZ Karlsruhe—Leibniz-Institut für Informationsinfrastruktur, Hermann-von-Helmholtz-Platz 1, Eggenstein-Leopoldshafen, Germany; 6 Institute of Computing, University of Campinas, Campinas, Brazil; 7 Department of Clinical Neuroscience, Karolinska Institutet, Solna, Sweden; 8 Swedish National Data Service, University of Gothenburg, Gothenburg, Sweden; 9 Cancer Research UK, London, UK; 10 German Human Genome-phenome Archive, DKFZ, Heidelberg, Germany; 11 University of Southampton, Southampton, UK; 12 INRAE, Direction des affaires juridiques, Paris, France; 13 University of Strasbourg, Bureau d’économie théorique et appliquée, Joint Unit 7522, Strasbourg, France

**Keywords:** Ethics, Economics

## Abstract

On 16 July 2020, the Court of Justice of the European Union issued their decision in the *Schrems II* case concerning Facebook’s transfers of personal data from the EU to the US. The decision may have significant effects on the legitimate transfer of personal data for health research purposes from the EU. This article aims: (i) to outline the consequences of the *Schrems II* decision for the sharing of personal data for health research between the EU and third countries, particularly in the context of the COVID-19 pandemic; and, (ii) to consider certain options available to address the consequences of the decision and to facilitate international data exchange for health research moving forward.

## Introduction

This paper considers the effects of the Court of Justice of the European Union’s (CJEU) *Schrems II* decision—on Facebook’s transfers of personal data (PD) from the EU to the US—on the international exchange of PD for health research, specifically in the context of the COVID-19 pandemic.

Processing data for health research often involves processing PD. To facilitate the exchange of PD across borders, states and supranational organisations have elaborated laws governing the conditions of exchange. The law elaborating the conditions governing such transfers from the EU is the general data protection regulation (GDPR) [1].

This law provides various justifications for the transfer of PD outside the EU. In practice, the law has generally functioned as a framework to transfer PD outside the EU for health research purposes—albeit with certain exceptions [2]. On 16 July 2020, the CJEU—the EU’s highest court—issued its decision in the *Schrems II* case concerning the law on transfers of PD from the EU to third countries [3].

In its decision, the court made several statements whose impact on international transfers of PD for health research purposes are likely to be significant—with the concept of ‘international transfers of PD for health research purposes’, we include all transfers, of all forms of PD, undertaken for the purpose of health research and exclude any transfers undertaken for other purposes.

In this article, we aim: (i) to outline the consequences of the case for the legitimate international exchange of PD for health research, specifically in the context of the COVID-19 pandemic; and (ii), to consider certain options to address the consequences of the case and to facilitate international data exchange moving forward.

We begin by describing the importance of international data exchange for health research (‘The importance of international data exchange in health research’). We continue by providing an overview of the justifications available under the GDPR for the legitimate transfer of PD for health research (‘The approach of the GDPR to international health research transfers’). We then provide a more detailed overview of the *Schrems II* case and how the decision impacts the transfer of EU PD for health research purposes (‘Schrems II: the case, the decision, and transfers of PD for health research’, ‘Broader consequences of Schrems II for transfers of PD for health research’ and ‘Limitations of Article 49 options’).

We then discuss two approaches to address the challenges posed to the international exchange of PD for health research by *Schrems II*. First, we highlight certain technological approaches (‘Technical measures to overcome challenges’). We also, however, highlight a need for caution in considering these technological approaches as providing holistic solutions (‘Limitations of technical measures to overcome challenges’).

Second, we discuss a policy solution in the form of international transfer agreements specific to transfers of PD for health research purposes. We argue that such agreements would be beneficial to international health research whilst, in certain cases at least, having little disadvantage to other pertinent EU or third country interests (‘Health research transfer agreements to overcome challenges’).

## The importance of international data exchange in health research

Health research is often an international endeavour. In this regard, at least three types of arguments outlining the importance of the international exchange of PD for health research can be put forward.

First, several kinds of health research depend completely on the international exchange of PD. Such research includes, for example, research into questions best addressed by comparing data from multiple populations in distinct regions and research which can only be conducted with access to PD sets located in multiple different countries. For example, international task forces/interest groups devoted to the study of rare diseases may be completely dependent on being able to pool PD across borders [4].

Second, the optimal conduct of health research requires that PD be as accessible as possible so research can be synthesised and built upon. In this regard: PD allows risks of bias to be assessed more thoroughly; PD permits flexible analysis, including reanalysis, through which research can be independently tested and verified with greater accuracy; and PD allows new scientific questions to be asked of existing data. For instance, the use of PD allows for investigations of interactions between interventions and patient-level characteristics not possible with other forms of data assemblies.

Finally, international data exchange serves ethical aims. It aids research resourcing and collaboration by reducing the resources required to collect PD. It maximises the contributions of individuals and institutions in the advancement of scientific knowledge. Further, data sharing brings researchers together into common projects and helps to break down the boundaries that impair research. International data exchange also facilitates the goal of equitable research, encouraging engagement, trust, transparency and the development of global capacity in research—see, for example, the CARE Principles [5].

The international exchange of PD for health research, however, is subject to legal conditions. Regarding transfers from the EU, these conditions are outlined in the GDPR.

## The approach of the GDPR to international health research transfers

Transfers of PD from the EU to third countries are only legitimate if based on a justification outlined in the GDPR. The aim of the justifications is to ensure that EU standards of data protection are maintained, in an ‘essentially equivalent’—the term used in the GDPR and in *Schrems II*— manner in third countries. The GDPR lists these justifications in Articles 44 to 49. Justifications are organised in a three-tier hierarchy [6]. In each tier, justifications relevant for health research transfers are identifiable.

### Tier 1

Adequacy decisions under Article 45. An adequacy decision is a decision from the European Commission that protection for PD in a third country is ‘essentially equivalent’ to that in the EU. With an adequacy decision in place, transfers can proceed freely—subject to conditions in the decision. The Commission has only issued few adequacy decisions [7]. It is unlikely this number will increase rapidly as the procedure can take several years [8].

In principle, adequacy decisions can provide a framework for transfers of PD for health research. Certain adequacy decisions, however, only apply to specific sectors in a third country and may thus be of limited use to researchers working outside these sectors. For example, the Canadian adequacy decision only concerns commercial organisations [9, 10].

### Tier 2

Transfers subject to appropriate arrangements. If an adequacy decision is not in place for a third country, transfers of PD for health research may be legitimised based on bi- or multilateral arrangements with recipients which assure the standard of protection elaborated by EU law. Article 46 outlines several relevant approaches: ‘a legally binding and enforceable instrument between public authorities’; ‘binding corporate rules’—agreements valid within a company or group of companies; ‘data protection clauses’—contractual clauses on data transfer; ‘codes of conduct’; ‘certification mechanisms’; and ‘provisions… inserted into administrative arrangements between public authorities’.

### Tier 3

Specific situations. If neither a tier 1 or tier 2 option is available, EU researchers may still transfer PD to third countries based on ‘specific situation’ justifications outlined in Article 49. Several such justifications are relevant for health research, including: if a subject ‘has explicitly consented to the proposed transfer’; ‘the transfer is necessary for important reasons of public interest’; ‘the transfer is necessary…to protect…vital interests’; and the transfer ‘is necessary for the purposes of compelling legitimate interests pursued by the controller…not overridden by the interests or rights and freedoms of the data subject’.

The general approach of the GDPR, however, is subject to judicial interpretation. In this regard, recently, an important decision was taken by the CJEU in the *Schrems II* case.

## *Schrems II*: the case, the decision, and transfers of PD for health research

On 16 July 2020, the CJEU handed down its judgment in *Data Protection Commissioner v. Facebook Ireland Ltd, Maximilian Schrems (Schrems II)*.

The case concerns questions as to how Facebook might legitimately transfer PD from the EU to the US. In particular, the case dealt with two forms of transfer justification: (i) the privacy shield agreement—an adequacy decision for US corporate actors; [11] and (ii) standard contractual clauses (SCCs)—a form of Article 46 contractual clause pre-approved by the European Commission [12, 13]. The key question addressed in the case concerned the legitimacy of using these justifications for transfers to the US given that the US legal system permits mass surveillance operations, in which US authorities can access, in bulk, PD transferred from the EU.

The CJEU found the US system did not provide a level of protection ‘essentially equivalent’ to that provided by the EU regarding US authorities’ powers to access, in bulk, EU citizens’ transferred PD. In particular, the CJEU highlighted two problems with the US system: (i) that intelligence agencies’ abilities to collect and analyse EU citizens’ PD was unnecessarily broad and included inadequate safeguards to protect rights—see, for example, para. 180; and (ii) the judicial review mechanisms for EU citizens did not meet the standards required by the EU Charter of Fundamental Rights—see, for example, para. 187.

The CJEU thus made two consequential pronouncements for health research transfers. First, the privacy shield adequacy decision was found to be illegitimate and was invalidated. Second, the SCCs outlined by the Commission are, in principle, still valid and useful to legitimate health research transfers from the EU to third countries but only if EU citizens’ rights in relation to the transfer(s) in question are provided with ‘essentially equivalent’ protection in the third country. Consequently, SCCs can only be used to legitimate health research transfers if: (i) a third country already provides ‘essentially equivalent’ protection; or (ii) supplementary measures assuring ‘essentially equivalent’ protection—contractual, technical, organisational, or other—can be put in place [14, 15].

Beyond the specific pronouncements made by the court, however, the decision also has much broader implications for international transfers of PD for health research.

## Broader consequences of *Schrems II* for transfers of PD for health research

Three broader consequences for health research transfers deserve further elaboration.

First, whilst *Schrems II* dealt specifically with privacy shield and SCCs, the logic of the decision applies to all another tier 2 transfer options for health research. In discussing SCCs, the CJEU was clear that to legitimately transfer PD from the EU, the law in a third country cannot function such that the standard of protection provided to EU citizens falls below the standard provided under EU law. In terms of their relationship with third-country law, all other tiers 2 justifications function comparably to SCCs. Thus, the basic requirements concerning SCCs and third-country laws apply to all other tiers 2 options.

The practical consequence of the above is that whilst previously researchers may have relied on uniform approaches —for example, uniform data transfer agreements—under tier 2 justifications, this is no longer possible. Researchers—or their host institutions—will now need to assess and confirm: (i) if a third country provides an ‘essentially equivalent’ standard of protection to that of EU law; and if not, (ii) whether supplemental safeguards can be implemented such that equivalent protection can be ensured in another way [14].

This will require researchers to expend supplemental resources in conducting the assessments needed to ensure legitimate transfers under tier 2 justifications. The precise scope and degree of the supplemental burden remains unclear. The burden may nevertheless—in some cases, at least—constitute an obstacle to health research transfers.^1^ Certain researchers may not possess the resources needed for assessments. Other researchers may be dissuaded from taking on the risk of assessments, given the potentially significant penalties applicable if transfers are considered illegitimate—including significant fines under Article 83(5) GDPR. Even researchers who have the necessary resources and are not averse to the risk will need to do cost-benefit analyses as to whether to divert resources to facilitate assessments.

Second, whilst the case specifically considered the US, the decision is also relevant for other third countries. Several other third countries have already been highlighted as potentially not providing ‘essentially equivalent’ protection. Some of these third countries engage in mass surveillance practices.^2^ Still, other third countries have been suggested to be problematic as a result of other local law, or the practical function of law in society [20].

Third, the case has reintroduced the problem of international data transfers into the sphere of active public and political debate. The underlying problems highlighted by the case are not new. Indeed, the problems highlighted by the CJEU concerning the privacy shield decision have been raised repeatedly over the past years [21]. Prior to the case, however, these problems had been addressed either with superficial legalistic solutions—such as that the use of SCCs alone provide suitable protection—or lacked substantive consideration. Following *Schrems II*, the underlying problems have been rudely exposed. They are no longer subject to superficial resolution and cannot be ignored.

One retort to our assertion that the consequences of the decision are significant for transfers of PD for health research might be: international transfer options outlined by non-US adequacy decisions and those in Article 49 (tier 3 options) are untouched by the decision. This retort is flawed. In relation to adequacy decisions, the flaw is that there are only a few decisions in force and these can thus only legitimate a limited set of transfers. In relation to Article 49 options, however, a deeper discussion is warranted.

## Limitations of Article 49 options

A look at the applicability conditions for Article 49 options in relation to health research reveals extensive limitations. Whilst full discussion is outside the scope of this paper—see Mitchell et. al for a deeper discussion—three types of limitation should be highlighted [22].

First, the applicability of several Article 49 justifications superficially relevant for health research transfers, is in fact unclear. This is the case, for example, in relation to Article 49(1)(d) justification ‘important reasons of public interest’. The EDPB recognises the justification may be used to legitimate certain transfers for health research in the context of the COVID-19 pandemic [23]. There remains a doubt, however, as to precisely which health research activities qualify as important public interests [24, 25].

Second, even when a justification’s applicability is clear, all Article 49 options are subject to general conditions limiting their utility for health research transfers. In particular, the EDPB highlights: ‘[tier 3 options] have to be interpreted in a way which does not contradict the… nature of [these options] as being exceptions from the rule’ [26]. This means Article 49 justifications should not justify run-of-the-mill transfers for health research or open, ongoing, sharing arrangements. The EDPB also highlights that: ‘Article 49 should never lead to a situation where fundamental rights might be breached’ [26]. This statement implies that even Article 49 justifications might not be used where third-country legal systems cannot guarantee ‘essentially equivalent’ protection.

Third, even when the applicability of an Article 49 justification is clear, and the general conditions for application are met, each justification is still subject to specific criteria limiting utility for health research transfers. To rely on Article 49(1)(a), for example, the GDPR requires a subject to be: ‘informed of the possible risks of such transfers…due to the absence of an adequacy decision and appropriate safeguards’. The EDPB suggests this condition implies a subject can only consent to a specific transfer to a third country where the risks associated with the transfer are clear in advance [22, 26].

In light of the prior analysis, an obvious question rears its head: how might the challenges posed by *Schrems II* for health research transfers be addressed moving forward? Here we suggest two approaches. Our first approach consists of technical measures.

## Technical measures to overcome challenges

There are technical measures that do not involve actual data transfer. As these measures do not involve the transfer of PD, they offer pathways to avoid the challenges posed by data protection transfer rules. There are perhaps three commonly used technologies that facilitate international access to health research data without transfer.

First: remote access via a thin client (data visitation). A thin client is usually a low-performance computer application that establishes a connection to a remote server. The thin client provides a window to the data stored at the source location whilst the data itself never leaves the source jurisdiction. Researchers access the data remotely to perform their analyses and may only retrieve results. Scholars, such as Mitchell et al., highlighted the argumentation in the CJEU *Lindqvist* case that the mere act of making data accessible may not constitute an international transfer under EU data protection law [22, 27].

Second: access via remote execution. In this model, data is not made available directly to researchers. Instead, researchers are given a codebook or synthetic version of the data, they can use to produce a code/script for the analysis to be performed. The results of the execution of the code/script are then assessed by the data hosts at the source location before being returned to the researchers. This removes the need for data to be transferred or for researchers to ever actually see the data.

Third: a hybrid model. Infrastructure solutions can offer health researchers greater flexibility compared to a remote execution model but without the need to see the data as required by data visitation via a thin client. Researchers are not able to see the underlying data, but receive anonymised results in real-time—anonymised data are not PD under the GDPR. Such infrastructure can also offer federated solutions.

Although technical measures are doubtless useful in facilitating international health research transfers where legal obstacles may make this difficult, each measure exhibits limitations. Accordingly, technical measures cannot provide holistic answers.

## Limitations of technical measures to overcome challenges

Three types of limitation to the utility of technical measures should be highlighted.

First, measures display scientific limitations. Approaches such as data visitation and remote execution show promise regarding analyses on single large data sets and for certain types of ‘second-level’ analysis. Other important research needs, however, cannot be met with these approaches. As outlined in ‘The importance of international data exchange in health research’, above, the availability of PD generally increases research efficiency and reduces bias. Data visitation and remote execution models do not permit PD to be exported and pooled in support of such ends.

Second, the measures display practical limitations. For example, users of data visitation or remote execution systems will require skills training and support to work in data holders’ systems. This requires resource investment and available expertise on the part of the recipient researcher. Equally, to ensure standards concerning the use of PD are maintained, technical measures require extensive governance and control systems. The construction and maintenance of systems are resource-intensive and may restrict possibilities for data analysis. Further, to enable reproducibility, there remains a need for guidelines and standards for reporting analyses performed under data visitation and remote execution models.

Third, the measures display legal limitations. Pertinent legal definitions remain vague and are subject to swift jurisprudential change—one example is the CJEU *Breyer* case in relation to the definition of PD [28]. There are thus limits to the conviction with which technical measures can be proposed as solutions to legal problems. Regarding data visitation, the problem appears with the legal concept of ‘transfer’. The *Lindqvist* case is now many years old and can be argued, as for example by Mitchell et al., to be out of step with more recent data protection thinking and jurisprudence [22]. The EDPB has even confirmed they see remote access as data transfer [15]. Regarding remote execution and hybrid models, the problem appears with the definition of PD and the degree to which synthetic data or anonymous data can be returned which are both scientifically useful and evade classification as PD [29].

The fact technical measures may be inadequate to resolve all issues connected with current EU data protection challenges to health research transfers engenders a need to look elsewhere for solutions. Our second approach thus consists of a policy proposal.

## Health research transfer agreements to overcome challenges

We propose considering the conclusion of unique bi- and multilateral agreements outlining principles specifically governing health research transfers.^3^ Such agreements could insulate transfers of PD for health research purposes from problematic, jurisdiction-specific, legislation—considering legal and practical limitations. In turn, such agreements could be concluded where general transfer agreements cannot be concluded and could continue functioning where general agreements fail. There are many objections which might be raised to this proposal.^4^ Nevertheless, we consider the idea as logical, interesting, and thus seek to open a discussion. Below, we sketch a four-pillar supporting argument.

First, the idea that transfers of PD for health research purposes should be considered as a unique form of international transfer should not be received as strange. There is a general recognition that the conflicts of interests engaged by PD processed for health research are distinct from those which characterise other—usually commercial or bureaucratic—data processing purposes. Processing of PD for health research purposes is thus often subject to separate legal and ethical conditions to those applicable to other processing purposes. This rationale is reflected in numerous international and national instruments. The rationale is even reflected in the GDPR. Against this background, why should international transfers of PD for health research purposes not be treated as unique forms of transfers?

Second, we would suggest that the negative impacts on EU and third countries’ non-research interests which, at least currently, would accrue from such agreements, would likely, in certain instances at least, be limited.^5^ For example, should the EU and the US conclude a bilateral health research-specific transfer agreement implementing limitations on US intelligence services’ activities necessary to meet EU standards, we would tentatively suggest the impact on US national security interests would be nominal. To our knowledge, PD transferred internationally for health research do not play a significant role in intelligence activities.^6^ We do not suggest that PD transferred internationally for health research should be considered ‘lower risk’ in relation to non-research processing. We simply aim to highlight that the interests underpinning non-research processing may, in certain instances at least, not suffer significantly from restricted access to this data.

Third, the benefit to health research may be significant. Under such agreements, researchers would be empowered to engage in the international exchange of PD for health research with a minimum of legal obstruction under EU data protection law. Equally, such agreements could mitigate against the risk that future jurisprudential developments concerning substantively different types of PD transfer might impact on the ability to engage in health research transfers—as in *Schrems II*. Further, such instruments could be tailored specifically to the needs of health research and thus provide researchers with clarity not necessarily present in generally applicable international transfer provisions.

Finally, in terms of the content of such instruments, health research is, usually, already subject to strict ethical approval. The ethical principles which govern such approvals already seek to protect the interests, and fundamental rights, of research subjects and to balance these against the potential benefit of health research to society as a whole. This scrutiny provides a precedent to limit and inform the policy measures suggested above.

## Conclusion

Transfers of PD for health research from the EU to third countries must be legitimated under one of the justifications in the GDPR. In principle, the GDPR offers a broad range of such justifications. In the recent *Schrems II* case, however, the CJEU made consequential pronouncements for the utility of these justifications for health research transfers.

Two pronouncements stand out. First, the CJEU invalidated the EU-US privacy shield adequacy decision. Second, the CJEU asserted that transfers based on bilateral agreements are only valid if an ‘essentially equivalent’ standard of protection to that offered under EU law can be ensured in the third country. This latter assertion has particularly wide-ranging implications for health research transfers.

From a legal perspective, the assertion is applicable to many of the justifications generally relevant for the legitimation of international transfers for health research under the GDPR. From a geographical perspective, the assertion is potentially relevant in relation to numerous third countries.

Certain GDPR justifications for legitimating international transfers for health research remain directly unaddressed in *Schrems II*. The case does not extensively discuss non-US adequacy decisions or the specific situation justifications in Article 49. The utility of these options for health research transfers, however, has limitations.

Moving forward, certain technical measures provide promising approaches to allow international health research involving EU PD to proceed despite legal obstacles. For example, in data visitation and remote execution models, PD is never transferred outside the EU. Such technical solutions, however, have their own limitations.

Policy measures may provide an alternative form of solution. In this regard, we would like to begin a discussion on the idea of health research-specific international agreements. Such agreements would be beneficial to international health research and, in certain cases at least, would likely have a little negative impact on pertinent non-research interests.

## Footnotes

1 The burden might be minimised by single assessments valid, or easily replicable, across multiple projects. The degree to which such assessments will be permissible under data protection law, however, and the degree to which they will be able to cover the range of forms of health research transfers and countries they may relate to, remains unclear. See [16].

2 See, for example, regarding Canada [17]. See, for example, regarding the UK [18]. On 31 January 2020, the UK withdrew from the EU, making the UK the third country. As of writing, EU–UK transfers continue largely as before under a ‘transition agreement’. This agreement is due to end, at the latest, on 30 June 2021. If no UK adequacy decision has been adopted by then the UK will be treated like any other third country—although the process toward adopting an adequacy agreement is now underway. See, for example, in relation to China [19].

3 In this article, we have focussed on transfers of personal data for health research purposes. There may be scope for discussing specific international transfer agreements in relation to other forms of transfer—including in relation to research transfers that contain PD relating to health, or PD from which health data may be inferred. Such discussions, however, are outside the scope of this article.

4 For example: the idea of such agreements may be criticised as idealistic and as distant from current political and legal realities; such agreements are unlikely to appear in the near future and cannot offer short-term solutions; numerous questions remain open concerning the substance of such agreements and the procedures for their conclusion; and such agreements would likely, often, face significant obstacles to conclusion.

5 We restrict our proposition to the current situation given the speed of technological advance and the ability for data sets to be subjected to advanced analytical processes—including AI—capable of drawing novel and unexpected inferences about individuals and populations.

6 We make this statement on the basis of the information we are aware of concerning the issue. However, the issue has not been a topic of extensive empirical research and certain law enforcement and intelligence activity is done without specific information ever being released to the public.
